# Reducing central line associated blood stream infections at intensive care unit

**DOI:** 10.1186/2047-2994-4-S1-P212

**Published:** 2015-06-16

**Authors:** HA Amer, Hala A Amer, Hind A Alzoman, Hassan Abdallah, Hanem Jomaa

**Affiliations:** 1 Infection Control Dept, King Saud Medical City, Riyadh, Saudi Arabia

## Introduction

Central Line Associated blood stream infections (CLABSI) is High Risk and High Volume problem, it prolongs hospitalization by mean of 7 days. Attribute cost per blood stream infection are estimated to be between 3700 $ and 29000$. Central line (CL) bundle is evidence-based practices which when implemented has demonstrated striking reduction in the rate of both central line related infections.

According 2012 Risk Assessment at king Saud Medical City, reduction of Central Line Associated Blood Stream Infection Rate in Adult ICU was a prioritized goal and planned to be achieved through implementation of central line bundle of care.

## Objectives

Reduce CLABSI rate at ICU by 20% annually staring in 2013

Achieve 90% CL Bundle compliance by end of 2014

## Methods

FOCUS PDCA Performance Improvement Project implemented. Team formulated of Infection control staff, ICU nurses and physicians and ICU director and the current practice of insertion and maintenance of Central Line catheter has been discussed

Improvment strategies include:

•Education about Central Line Bundle of Care for ICU staff

•Make the supply available in ICU to maintain bundle of care for central line (e.g. full size drape, CHG applicator and CHG impregnated dressing).

•Checklist developed for bundle variables and placed at patient Medical Record

•Supervised central line insertions by secret observer

•Nurses are empowered to stop any central line insertion in case of any breach of infection control guidelines and practices.

•Replaced povidone iodine with CHG which is superior.

•Encourge strict adherence to hand hygiene practices.

•Discouraged the use of suboptimal site such as the femoral by means of email alerts

•Communication with emergency room team to reduce the femoral central lines coming from E.R.

•Replacing CVL inserted under emergent situation within 48 hours

•Audit sheet used to calculate bundle compliance

•Doing daily bathing with CHG for the patients.

## Results

40% reduction of CLABSI occured within 2 years (from 11.3 in 2012 to 6.8 in 2014)

Bundle compliance ranges between 84% to 97% during 2014

Femoral insertions declines from 10/month to 1-3/month in 2014

## Conclusion

Involving process owner champions, consistent availability and accessibility of required supply for Central Line Bundle and continuous Staff education and monitoring with feedback for caregivers are effective strategies to reduce CLABSI

## Disclosure of interest

None declared.

